# Associations between peripheral blood whole-genome DNA methylation and obesity in young Chinese males

**DOI:** 10.20945/2359-4292-2026-0060

**Published:** 2026-06-08

**Authors:** Meidan Nong, Haiou Lu, Maolin Zhao, Lihong Xiao

**Affiliations:** 1 General Medicine Department, Liuzhou People’s Hospital, Liuzhou City, Guangxi Zhuang Autonomous Region, China

**Keywords:** Obesity, methylation, methyltarget technology, *LGI1* gene, body mass index

## Abstract

**Objective:**

To explore the associations between peripheral blood whole-genome DNA methylation and obesity in young Chinese males.

**Subjects and methods:**

Four young Chinese males aged 18-25 years with obesity (body mass index [BMI] ≥ 30 kg/m^2^) from Liuzhou Peoples Hospital Health Management Center and 3 age-matched healthy controls with average weights were included. Methylation sequencing was conducted using whole-blood DNA extraction and an Illumina 850K methylation chip, followed by gene functional annotation, pathway enrichment analysis, and differential analysis using bioinformatics tools.

**Results:**

MethylTarget technology was utilized for the methylation sequencing of 35 patients with obesity and 34 healthy controls, revealing 15 differential sites, two differential regions, and three differential genes. The differential regions included cg08667244 (site region 1stExon) and cg13928759 (site region TSS200, p < 0.05), with the differential genes being cg08667244 (corresponding to iodothyronine deiodinase 3, DIO3), cg13928759 (corresponding to leucine-rich glioma inactivated 1, LGI1), and cg18770216 (corresponding to integrin beta 5, ITGB5, p < 0.05). Further analysis revealed a negative correlation between cg13928759 methylation (*LGI1* gene) and body mass index (R = -0.25; P = 0.04).

**Conclusion:**

This study revealed 15 differential sites, two differential regions, and three differential genes between young individuals with obesity and healthy controls, among which cg13928759 methylation (LGI1 gene) was negatively correlated with body mass index. These findings suggest that DNA methylation may contribute to obesity development, with LGI1 methylation serving as a potential biomarker.

## INTRODUCTION

Obesity among young males is a growing global concern, with a steady increase in cases occurring in recent years (^1^). World Health Organization (WHO) reports that more than 400 million people worldwide are battling obesity, and a significant number of them are young males. This condition involves more than carrying extra weight (^2^). It is linked to serious health issues, such as heart disease and diabetes, and significantly affects individuals’ quality of life and health (^3^). However, our understanding of what causes obesity in young males, especially from an epigenetic perspective, remains limited (^4^). Epigenetics, particularly DNA methylation, plays a crucial role in controlling the expression of metabolism-related genes, including those involved in fat synthesis, energy use, and hormone levels, all of which are vital for managing weight and energy balance (^5^,^6^). Addressing obesity in young males requires a deeper understanding of how methylation affects gene expression and obesity (^7^). Exploring this relationship could unlock new ways to manage or even prevent obesity, not just in young males but potentially in wider populations (^8^). Simplifying complex scientific concepts and focusing on actionable insights can make discussions of obesity management more engaging and practical for a broader audience, thereby encouraging positive lifestyle changes and greater awareness of this global health challenge.

Currently, interventions for obesity mainly include dietary control, exercise, and drug therapy. However, these interventions have limitations in practical applications, such as limited long-term effectiveness and treatment tolerance; thus, further research and exploration are needed (^9^-^11^). This study aimed to address two fundamental questions: (^1^) What genome-wide DNA methylation signatures distinguish young males with obesity from lean young males in a Chinese population? (^2^) How do these epigenetic modifications correlate with clinical obesity indicators, such as body mass index (BMI)? The aim of this study was to identify differentially methylated regions (DMRs) in the promoter and gene body regions of metabolism-related genes using MethylTarget sequencing. We focused on CpG sites within the TSS200, 1stExon, and 5’UTR regions to explore their associations with BMI and obesity-related metabolic traits in young males. Additionally, this study extensively employed bioinformatics tools to analyze methylation data to identify core methylation genes and pathways related to obesity, thereby offering new insights and strategies for preventing and treating obesity in the future.

## SUBJECTS AND MATERIALS

This cross-sectional study was approved by the Ethics Committee of Liuzhou People’s Hospital (KY2021-019-01). Participants provided written informed consent to participate in this study.

### Inclusion and exclusion criteria

The subjects in the obesity group included in this study were Chinese men aged 18-25 years who met the diagnostic criteria for simple obesity of the WHO (BMI ≥ 30 kg/m^2^, excluding secondary factors such as endocrine disorders and genetic syndromes) and had no history of chronic metabolic diseases, such as diabetes, hypertension, or dyslipidemia. These subjects had not used any drugs that affect weight or metabolism in the past six months. Chinese men with an age difference of no more than 2 years and a BMI within the normal range (18.5-24.9 kg/m^2^) were included in the control group; a family history of obesity among their immediate family members was not required. The common exclusion criteria for the two groups included the presence of acute or chronic inflammatory diseases (such as autoimmune diseases or a recent history of infection); previous bariatric surgery or bariatric intervention; the use of drugs that affect DNA methylation, such as glucocorticoids or antipsychotic drugs; smoking more than 5 cigarettes per week or an alcohol intake > 140 grams; and participation in other clinical trials within the past 3 months.

### Whole-blood genomic methylation sequencing method

DNA was extracted from whole-blood samples of each participant using commercial extraction kits (such as Qiagen or Omega Biotek), followed by quality and concentration testing to ensure the integrity and purity of the DNA. DNA libraries were prepared for methylation sequencing, and high-throughput sequencing of methylation sites was performed using Illumina 850K methylation chips to ensure coverage of methylation information across the entire genome. Methylation sequencing data were aligned, and methylation sites were identified using software such as Bismark or Bowtie, while low-quality sequences and polymerase chain reaction (PCR) duplicates were filtered out. Standardized beta values were subjected to singular value decomposition (SVD) to assess the correlation strength between each component and the factors in the data. These results reflected the existence of batch effects to some extent. If batch effects were found, batch effect correction was performed. The methylation result files for all samples were converted to bedGraph format files to visualize specific sites or regions of interest using Integrative Genomics Viewer (IGV) software (http://www.broadinstitute.org/igv/home).

### Whole-genome methylation differential analysis

The Chip Analysis Methylation Pipeline (ChAMP) package was employed for differential methylation analysis using statistical tests such as the T test and Wilcoxon rank sum test. Multiple comparison corrections ensured the significance of the differential methylation sites.

### Analysis of obesity-related methylation sites/regions

MethylTarget technology was used for targeted methylation site sequencing and to quantify methylation level differences. Correlation analysis revealed associations between differential methylation sites/regions and BMI.

### Gene functional annotation and pathway enrichment analysis

The methylation data were processed for gene functional annotation using Bioconductor’s methyl Kit and ChAMP packages. This processing included the localization of methylation sites on the genome and correlation analysis with gene function. Methylation site functions in the genome were evaluated on the basis of location information, such as whether the sites were located in promoter regions, gene bodies, 3’UTRs, or 5’UTRs. Further analysis was conducted to determine the enrichment of differential methylation sites in biological functions and pathways using tools such as Database for Annotation, Visualization and Integrated Discovery (DAVID) and Metascape, which included Gene Ontology (GO) and Kyoto Encyclopedia of Genes and Genomes (KEGG) pathway enrichment analysis. To determine the differential methylation sites related to obesity, the biological significance of the enriched pathways was analyzed, including whether they were involved in lipid metabolism, insulin secretion, and other obesity-related processes.

### Data analysis

All statistical analyses were performed using R version 4.3.2 (R Foundation for Statistical Computing). Differential methylation analysis was conducted through a two-stage approach: 1) genome-wide screening using linear regression models adjusted for age and batch effects (limma package), and 2) targeted validation using paired T tests for confirmed CpG sites. The significance threshold was set at p < 0.05 for the initial discovery phase analyses, with false discovery rate (FDR) correction (Benjamini-Hochberg method) applied for multiple comparisons (an FDR < 0.15 was considered suggestive, and an FDR < 0.05 was deemed significant). Effect sizes were calculated using Cohen's d for differences in methylation beta values (|d| > 0.5 was considered a moderate effect). For correlation analyses between methylation levels and BMI, Pearson’s correlation coefficients (for normally distributed data, Shapiro-Wilk test p > 0.05) or Spearman's rank correlation (for non-normally distributed data) were employed. Power analysis indicated 80% power to detect methylation differences with an effect size of ∆β = 0.2 at α = 0.05 (two-tailed) given our sample size (G*Power 3.1.9.7).

## RESULTS

### Analysis of sample size and basic characteristics of the population

**Table 1** summarizes the baseline characteristics of the discovery cohort (4 patients with obesity vs. 3 controls). Compared with the control group, the obesity group had significantly greater BMI (40.1 ± 1.7 vs. 21.8 ± 1.4 kg/m^2^, p = 0.00) and body weight (118.2 ± 14.0 vs. 64.6 ± 6.2 kg, p = 0.01), while no differences were observed in age (18.5 ± 1.0 vs. 18.3 ± 0.6 years, p = 0.79) or height (171.5 ± 11.4 vs. 172.3 ± 10.8 cm, p = 0.93). These findings were validated in the larger cohort (**Table 2:** 35 patients with obesity vs. 34 controls), where the obesity group again had elevated BMI (34.2 ± 2.1 vs. 20.5 ± 1.5 kg/m^2^, p < 0.001) and weight (102.0 ± 11.2 vs. 58.3 ± 6.0 kg, p < 0.001), with comparable age (18.6 ± 0.7 vs. 18.4 ± 0.7 years, p = 0.27) and height (172.8 ± 7.3 vs. 168.4 ± 6.6 cm, p = 0.01). Both cohorts demonstrated robust anthropometric matching, minimizing confounding effects in subsequent methylation analyses. Data are presented as the mean ± SD; p values are derived from Student’s T test (p < 0.05). Gene functional annotation and KEGG pathway enrichment analysis were conducted using R to select core methylation genes and sites related to obesity. Thirty-five young male patients with obesity and 34 age-matched healthy males with average weights were selected, and whole-blood DNA was extracted for targeted region methylation sequencing and methylation level detection. The two groups differed significantly in terms of the leading observational indicators, namely, BMI, weight, and height, while individuals with hypertension, diabetes, cardiovascular diseases, severe liver or kidney dysfunction, and chronic diseases were excluded. No significant differences were observed in the other data (**Table 2**).

**Table 1:** Baseline characteristics of the discovery cohort (4 patients with obesity vs. 3 controls)

Age (years)		Height (cm)		Weight (kg)		BMI (kg/m^2^)		
Normal	Obesity	Normal	Obesity	Normal	Obesity	Normal	Obesity	
N (3)	N (4)	N (3)	N (4)	N (3)	N (4)	N (3)	N (4)	
18-25	18-25	172.33	171.50	64.57	118.15	21.76	40.11	Average
0.577	1.000	10.786	11.382	6.192	13.956	1.367	1.668	SD

**Table 2:** Baseline characteristics of the larger cohort (35 patients with obesity vs. 34 controls)

Age (years)		Height (cm)		Weight (kg)		BMI (kg/m^2^)		
Normal	Obesity	Normal	Obesity	Normal	Obesity	Normal	Obesity	
N (34)	N (35)	N (34)	N (35)	N (34)	N (35)	N (34)	N (35)	
18-25	18-25	168.38	172.76	58.27	102.02	20.53	34.20	Average
0.657	0.736	6.629	7.272	6.004	11.204	1.451	2.066	SD

### Whole-genome methylation differential analysis

**Figure 1:**
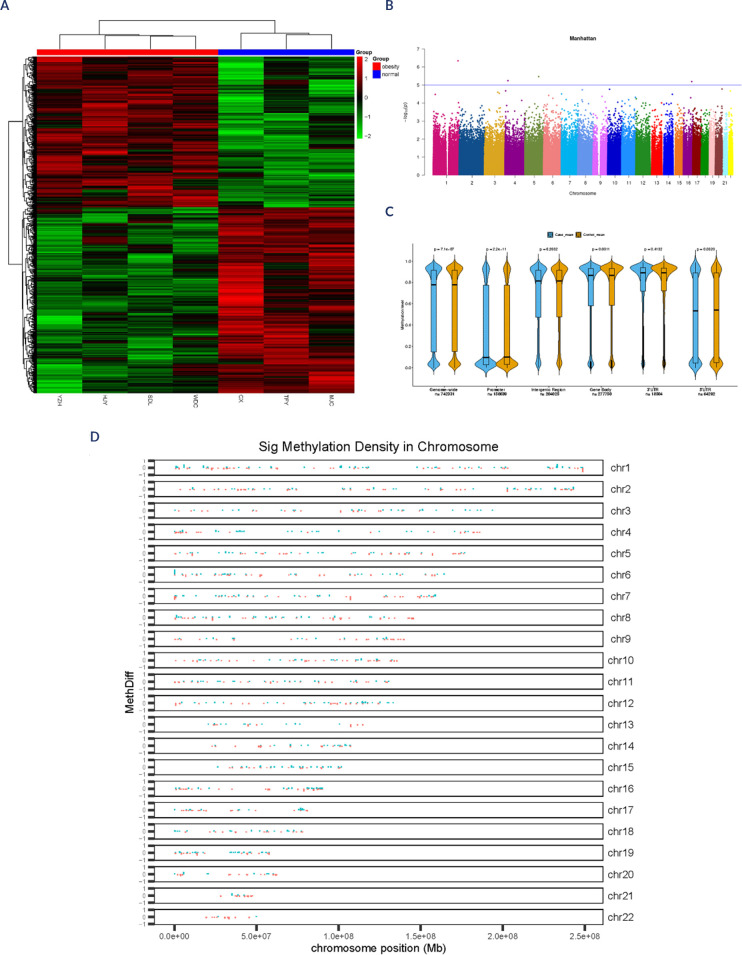
Genome-wide methylation profiling of obesity-associated CpG sites. (**A**) Hierarchical clustering of 7 samples (4 individuals with obesity: O1-O4; 3 controls: C1-C3) based on methylation levels at 23 differentially methylated CpG sites (∆β > 0.2, p < 0.05). Clustering was performed using the Pearson correlation distance with complete linkage. The heatmap colors represent beta values: hypomethylated (blue, β < 0.3), intermediately methylated (white, 0.3 ≤ β ≤ 0.7), and hypermethylated (red, β > 0.7). Columns are annotated with group labels (obesity vs. control). (**B**) Distribution of methylation regions across genomic features (promoter, gene body, 3’UTR, 5’UTR, and intergenic regions). (**C, D**) IGV visualization of two significant differentially methylated regions (cg08667244 in DIO3 and cg13928759 in LGI1) mapped to their respective genomic loci.

For the obesity group, comprising four cases, and the healthy group, comprising three cases, whole-blood DNA 850K methylation sequencing was performed, and 734,163 methylation sites were detected per sample. Using the ChAMP package in R, differential methylation analysis was conducted on the methylation sites between the obesity and healthy groups. The differential methylation results are displayed as a heatmap, which shows the expression patterns of the genes containing differentially methylated sites in the grouped samples (**Figure 1A**). The entire genome methylation sequence regions analyzed included the genome-wide region, promoter, intergenic region, gene body, 3’UTR, and 5’UTR (**Figure 1B**), with 23 differentially methylated regions identified. Differentially methylated sites or regions were mapped to the genome, and the differentially methylated genes were further analyzed (**Figure 1C, D**; p < 0.05).

### Enrichment analysis of whole-genome methylation sites/region-associated genes

During the GO functional analysis, 309 biological processes (BPs), 99 cellular components (CCs), and 50 molecular functions (MFs) had corrected P < 0.05 among the differential methylation sites and regions. The top 10 functions of these sites are oligosaccharide binding, ligand-gated calcium channel activity, G-protein-coupled receptor binding, calcium release channel activity, phosphatidylinositol-4,5-bisphosphate binding, phosphatidylinositol bisphosphate binding, and protein binding, which play crucial roles in regulating obesity metabolism pathways. The focus of methylation binding regions is on regulating enzyme activity, carbon-nitrogen bond formation, protein serine/threonine/tyrosine kinase activity, intramolecular oxidoreductase activity, peptide antigen binding, epidermal growth factor receptor binding, fibroblast growth factor binding, cellular metabolic complex salvage, and cellular modified amino acid metabolism processes. These functions, along with pathway enrichment analyses, including cysteine and methionine metabolism, cortisol synthesis and secretion, parathyroid hormone synthesis and action, Rap1 signaling, aldosterone synthesis and secretion, and sphingolipid signaling pathways, among others, contributed to the enrichment of multiple lipid metabolism regulation and obesity-related signaling pathways. We identified thirty KEGG pathways and GenBank pathways associated with obesity on the basis of the enrichment results. Furthermore, we selected thirty-five obesity-related methylation sites and their associated genes (**Table 3**).

**Table 3:** GO and KEGG enrichment analysis of obesity-related methylation sites

CpG	CHR	Gene	Group	GO-BP	GO-MF	GO-CC	KEGG	FoldChange	P.Value
cg05872034	1	NPHP4	Body	GO:0098609,cell-cell adhesion	GO:0005515,protein binding	GO:0005813,centrosome		2.24	0.03
cg00371383	1	FMN2	Body	GO:0015031,protein transport	GO:0003779,actin binding	GO:0030659,cytoplasmic vesicle membrane	hsa04320 Dorso-ventral axis formation	1.23	0.00
cg21379113	1	AKT3	Body	GO:0006468,protein phosphorylation	GO:0004674,protein serine	GO:0016020,membrane	hsa05222 Small cell lung cancer	0.73	0.05
cg09858955	2	VRK2	5’UTR	GO:0006468,protein phosphorylation	GO:0004674,protein serine	GO:0005737,cytoplasm		1.30	0.00
cg11765003	2	NHEJ1	5’UTR	GO:0006310,DNA recombination	GO:0005515,protein binding	GO:0005634,nucleus	hsa03450 Non-homologous end-joining	1.27	0.01
cg16658156	2	HTR2B	5’UTR	GO:0006182,cGMP biosynthetic process	GO:0005096,GTPase activator activity	GO:0030054,cell junction	hsa04020 Calcium signaling pathway	1.28	0.04
cg05171197	2	HDAC4	Body	GO:0006954,inflammatory response	GO:0005515,protein binding	GO:0043234,protein complex	hsa04371 Apelin signaling pathway	1.16	0.00
cg25117092	3	MED12L	Body	GO:0006351,transcription, DNA-templated|	GO:0008013,beta-catenin binding	GO:0016592,mediator complex	hsa04919 Thyroid hormone signaling pathway	1.37	0.00
cg03199564	4	CYTL1	1stExon	GO:0007165,signal transduction	GO:0005102,receptor binding	GO:0005615,extracellular space		0.43	0.00
cg18770216	3	ITGB5	Body	GO:0007160,cell-matrix adhesion|	GO:0005515,protein binding	GO:0009986,cell surface	hsa04145 Phagosome	0.7	0.03
cg05688588	4	PI4K2B	Body	GO:0007032,endosome organization	GO:0005524,ATP binding	GO:0005768,endosome	hsa01100 Metabolic	2.15	0.002
cg20701305	4	STIM2	Body	cellular calcium ion homeostasis	|GO:0005515,protein binding	GO:0005783,endoplasmic reticulum	hsa04020 Calcium signaling pathway	1.62	#####
cg16006841	5	RGS14	Body	GO:0000278,mitotic cell cycle	GO:0005515,protein binding	GO:0030054,cell junction	hsa04015 Rap1 signaling pathway	1.63	0.04
cg22996185	6	SYNE1	Body	GO:0007030,Golgi organization	GO:0005515,protein binding	GO:0005794,Golgi apparatus		3.52	0.01
cg05977964	7	ADAP1	TSS200	GO:0007166,cell surface receptor signaling pathway	GO:0005515,protein binding	GO:0005737,cytoplasm		0.79	0.01
cg10020767	7	CREB5	Body	GO:0006366,transcription from RNA polymerase II promoter	GO:0005515,protein binding	GO:0005634,nucleus	hsa04152 AMPK signaling pathway	1.20	0.01
cg04790662	8	PAG1	5’UTR	GO:0007165,signal transduction	GO:0005515,protein binding	GO:0005886,plasma membrane		2.64	0.01
cg22137815	8	SDC2	Body	GO:0044267,cellular protein metabolic process	GO:0005515,protein binding	GO:0005788,endoplasmic reticulum lumen	hsa05418 Fluid shear stress and atherosclerosis	1.53	0.03
cg01284841	10	CDH23	Body	GO:0006816,calcium ion transport	GO:0005515,protein binding	GO:0005886,plasma membrane		1.25	2E-04
cg13928759	10	LGI1	TSS200	GO:0008283,cell proliferation	GO:0005515,protein binding	GO:0030054,cell junction		1.21	#####
cg11430259	10	FGFR2	5’UTR	GO:0043410,positive regulation of MAPK cascade	GO:0005515,protein binding	GO:0005794,Golgi apparatus	hsa04010 MAPK signaling pathway	1.45	0.04
cg17820828	11	KCNQ1	Body	GO:0071805,potassium ion transmembrane transport	GO:0005515,protein binding	GO:0005783,endoplasmic reticulum	hsa04974 Protein digestion and absorption	0.70	#####
cg18427217	11	SHANK2	3’UTR	GO:0060291,long-term synaptic potentiation	GO:0005515,protein binding	GO:0030054,cell junction	hsa04724 Glutamatergic synapse	1.19	0.002
cg01039401	12	TEAD4	5’UTR	GO:0006357,regulation of transcription from RNA polymerase II promoter	GO:0005515,protein binding	GO:0005634,nucleus	hsa04390 Hippo signaling pathway	0.82	#####
cg17640061	12	SYT1	Body	GO:0051260,protein homooligomerization	GO:0005515,protein binding	GO:0005794,Golgi apparatus	hsa04721 Synaptic vesicle cycle	2.42	0.02
cg15319704	13	KLF5	TSS1500	GO:0008284,positive regulation of cell proliferation	GO:0005515,protein binding	GO:0005794,Golgi apparatus		2.50	0.04
cg08667244	14	DIO3	1stExon	GO:0042404,thyroid hormone catabolic process	GO:0004800,thyroxine 5’-deiodinase activity	GO:0010008,endosome membrane	hsa04919 Thyroid hormone signaling pathway	0.81	#####
cg18266472	15	LINGO1	TSS200	GO:0007165,signal transduction	GO:0005515,protein binding	GO:0005578,proteinaceous extracellular matrix		2.82	0.003
cg03660162	16	WWOX	Body	GO:0072332,intrinsic apoptotic signaling pathway by p53 class mediator	GO:0046983,protein dimerization activity	GO:0005794,Golgi apparatus		0.58	#####
cg19944656	17	TIMP2	Body	GO:0045930,negative regulation of mitotic cell cycle	GO:0005515,protein binding	GO:0005578,proteinaceous extracellular matrix		3.29	0.002
cg03181524	19	RYR1	Body	GO:0051289,protein homotetramerization	GO:0005515,protein binding	GO:0005790,smooth endoplasmic reticulum	hsa04713 Circadian entrainment	1.53	0.02
cg12602339	20	DEFB126	TSS1500	GO:0042742,defense response to bacterium		GO:0005576,extracellular region		1.17	3E-04
cg17036908	20	CDH4	1stExon	GO:0007155,cell adhesion	GO:0005509,calcium ion binding	GO:0005886,plasma membrane	hsa04514 Cell adhesion molecules (CAMs)	1.26	2E-04
cg01531431	20	PPDPF	TSS1500	GO:0031017,exocrine pancreas development				0.84	#####
cg11287055	21	DSCR3	Body	GO:0006886,intracellular protein transport	GO:0006886,intracellular protein transport	GO:0005634,nucleus		2.30	0.006

### Analysis of differential methylation sites/regions associated with obesity

**Supplementary Figure 1:**
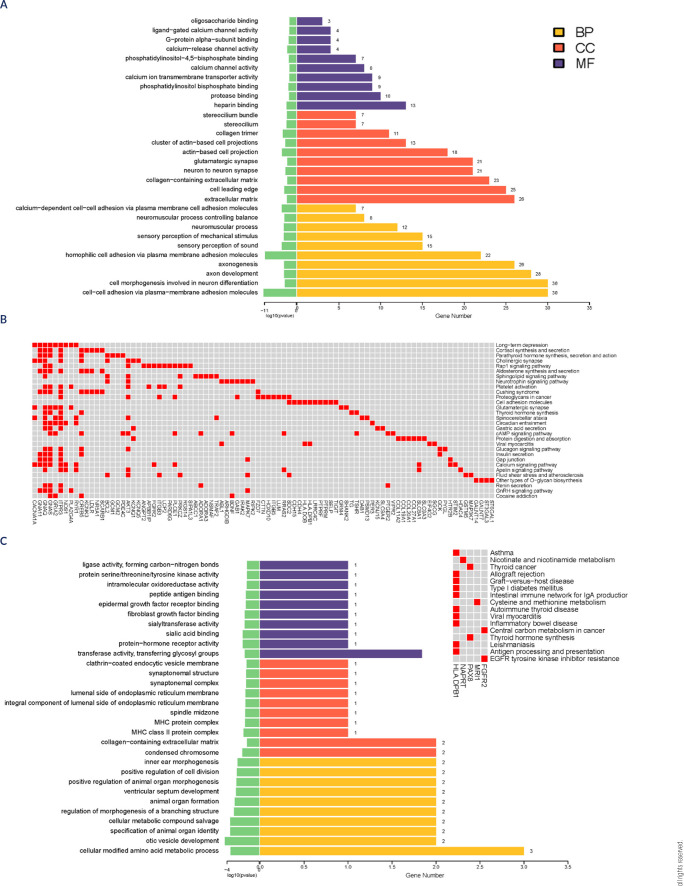
Genome-wide methylation site/region-related gene enrichment analysis. (**A**) GO functional enrichment analysis of differential methylation sites and regions; (**B**) KEGG signaling pathway enrichment analysis of differential methylation sites and regions; (**C**) GO and KEGG enrichment analysis of methylation binding regions.

Using MethylTarget technology, methylation sequencing and methylation level detection were performed for target methylation sites, methylation regions, and target genes. Fifteen differential methylation sites were identified between the obesity group (35 cases) and the healthy group (34 cases), along with two differential methylation regions (cg08667244 in the 1stExon region and cg13928759 in the TSS200 region; p < 0.05) and three differential methylation genes (cg08667244 corresponding to iodothyronine deiodinase 3 (DIO3), cg13928759 corresponding to leucine-rich glioma inactivated 1 (LGI1), and cg18770216 corresponding to integrin subunit Beta 5 (ITGB5); p < 0.05). Further analysis revealed a negative correlation between the methylation level of cg13928759 (LGI1 gene) and BMI, with R = -0.25 and P = 0.04 (**Supplementary Figure 1, Table 4**).

**Table 4:** Results of differential analysis of obesity-related methylation sites

Target	Position	Type	P-value (Ttest)	P-value (Utest)	P-value (Logistic)	FP Mean	FP Median
cg01531431_38_N	57	CG	0.043942892	0.043762878	0.05108085	0.404772099	0.393162393
cg03199564_9	125	CG	0.024366003	0.020188162	0.031946353	0.083718639	0.083509514
cg03199564_9	155	CG	0.015856406	0.021555529	0.021661689	0.037006892	0.034587996
cg05649108_34	29	CG	0.007584019	0.006146756	0.01204122	0.180212038	0.170242215
cg05649108_34	31	CG/cg02345961	0.022485902	0.032748724	0.028766069	0.12788573	0.125721616
cg05649108_34	86	CG	0.00444197	0.002957483	0.007497966	0.312951408	0.305280528
cg05649108_34	105	CG	0.036562359	0.028295131	0.042942078	0.224436596	0.215355805
cg05649108_34	159	CG	0.045160769	0.024057175	0.049919535	0.122291469	0.112239444
cg05649108_34	164	CG	0.023016181	0.023278119	0.027925446	0.109088574	0.099491238
cg13928759_23	102	CG/cg13928759	0.008878135	0.021555529	0.013841437	0.589745522	0.591511692
cg18266472_31	27	CG	0.053375892	0.022268526	0.058380429	0.012734543	0.012524085
cg18266472_31	46	CG	0.025650349	0.041384559	0.029540485	0.017254492	0.016991041
cg18266472_31	90	CG	0.028353871	0.012990731	0.035837213	0.019125757	0.018391889
cg18427217_26_N	59	CG	0.039450907	0.033537693	0.045690323	0.496114202	0.492574865
cg20701305_12_N	96	C.G./cg20701305	0.040809511	0.055205584	0.05153688	0.260917881	0.262063228

## DISCUSSION

The occurrence of adolescent obesity involves a variety of complex mechanisms and factors. Genetic, lifestyle, environmental, and psychological factors may play essential roles in obesity (^12^-^14^). In addition, metabolic abnormalities, brain appetite regulation centers, and inflammatory responses are closely related to adolescent obesity (^15^). Some adolescent individuals with obesity may have metabolic abnormalities, such as insulin resistance and thyroid dysfunction, which can affect energy balance and fat metabolism, leading to obesity (^16^). Furthermore, the regulation of food intake by appetite control centers in the brain (such as the hypothalamus) is also associated with obesity (^17^). Abnormal signaling of some hormones (such as insulin and leptin) may affect satiety and appetite control, affecting weight regulation (^18^).

Additionally, studies have shown a close relationship between inflammation and obesity. In obesity, the levels of inflammatory factors increase in adipose tissue, which may lead to insulin resistance, increased adipocyte numbers, and obesity-related diseases (^19^). These factors interact, leading to an imbalance between energy intake and expenditure, resulting in excessive fat storage and obesity.

In this study, MethylTarget technology was used to conduct methylation sequencing analysis on adolescent patients with obesity, and differential methylation sites and genes associated with obesity were discovered. The differential methylation regions included cg08667244 (site region 1stExon) and cg13928759 (site region TSS200), and the corresponding differential genes included cg08667244 (corresponding gene DIO3), cg13928759 (corresponding gene LGI1), and cg18770216 (corresponding gene ITGB5). Further analysis revealed that the methylation level of cg13928759 (corresponding gene LGI1) was negatively correlated with BMI.

The brain and nervous system are essential for weight regulation, as they regulate appetite, metabolic rate, and fat distribution, which influence weight maintenance. The regulation of hunger and satiety, maintenance of energy balance, and coordination of food intake and expenditure are critical mechanisms through which the brain and nervous system regulate weight (^20^). Neural circuits include the hypothalamic-pituitary-adrenal (HPA) axis and the hypothalamic-pituitary-thyroid (HPT) axis, which may be regulated by endocrine factors and neurotransmitters, thereby affecting appetite, metabolism, and fat distribution and ultimately affecting the occurrence and development of obesity (^21^). LGI1 is a gene-encoding protein that is usually associated with neuronal activity and neurological diseases (^22^). Recent studies have suggested that LGI1 may also be associated with energy metabolism and weight control (^23^). Our research revealed a negative correlation between the methylation level of LGI1 and BMI, suggesting that LGI1 may play a role in the occurrence and development of obesity. Similarly, genes such as DIO3 and ITGB5 may also affect obesity through mechanisms such as the regulation of thyroid hormone metabolism, cell signaling, or inflammatory responses.

Previous studies have reported various associations between methylation and obesity-related factors. Compared with these studies, our findings on the differential methylation sites cg08667244, cg13928759, and cg18770216 and their corresponding genes DIO3, LGI1, and ITGB5 show both similarities and differences.

In terms of DIO3, several prior investigations have indeed demonstrated its link to energy metabolism, which aligns with our results. For instance, alterations in DIO3 methylation status influenced thyroid hormone-mediated energy expenditure in adipose tissue. Similar to these findings, our research also revealed that methylation of the DIO3 gene, specifically at the cg08667244 site, is associated with obesity, suggesting its potential role in disrupting energy balance. However, differences in the specific methylation sites identified were detected. While our study pinpoints cg08667244 as a key differential site, other studies may have focused on different regions of the DIO3 gene. Additionally, the regulatory mechanisms may vary. In our study, we hypothesized that methylation at cg08667244 could directly affect the binding of transcription factors to the DIO3 promoter region, thereby modulating gene expression and subsequent energy metabolism. In contrast, previous research might have proposed alternative regulatory pathways, such as post-translational modifications of the DIO3 protein.

With respect to LGI1, most prior research has focused predominantly on its role in neurological diseases. Our study offers a novel perspective by revealing its potential connection with obesity through the negative correlation between the methylation level of cg13928759 (LGI1 gene) and BMI. Although the exact mechanism remains unclear, the role of LGI1 in neuronal activity might indirectly influence appetite regulation or energy expenditure-related neural circuits, which are crucial for maintaining a healthy body weight. These findings expand the functional understanding of LGI1 beyond the neurological realm.

With respect to ITGB5, few studies have explored its relationship with obesity. Existing research on ITGB5 has focused mainly on its function in cell adhesion, migration, and tumor metastasis. For example, one study reported that ITGB5 is involved in the invasion and metastasis of cancer cells through the regulation of cell-extracellular matrix interactions. Our study is among the first to identify cg18770216 as a regulator of obesity. We propose that methylation in the ITGB5 gene may impact the inflammatory response and differentiation of adipocytes, which are key processes in obesity development. These findings provide new insights into the role of ITGB5 in the context of obesity-related pathophysiology.

Energy metabolism refers to the processes of energy synthesis, utilization, and storage in the body. In obesity, energy metabolism may be dysregulated, leading to an imbalance between energy intake and expenditure (^24^). Obesity is often accompanied by an imbalance in energy metabolism, such as excessive food intake and insufficient physical activity, which can lead to an energy surplus (^25^). In addition, the abnormal secretion or action of metabolic regulatory factors, such as insulin and thyroid hormones, may affect energy metabolism, thereby promoting the occurrence of obesity (^26^). This study revealed that the obesity-related differential gene cg08667244 (corresponding gene DIO3) has a potential influence on the obesity process. DIO3 encodes type 3 iodothyronine deiodinase, and the methylation status of the DIO3 gene is closely related to energy metabolism (^27^), suggesting that DIO3 may participate in obesity by affecting thyroid hormone levels and metabolic regulation.

Fat metabolism encompasses fat synthesis, breakdown, and storage processes. In cases of obesity, this metabolism can become imbalanced, leading to excessive fat accumulation and obesity development. Key characteristics of obesity include an increase in both the number and size of fat cells, possibly owing to imbalances in energy intake and release, resulting in heightened fat storage (^28^). Moreover, secretions from fat cells, such as inflammatory factors, may influence the body’s metabolic state, impacting the onset and progression of obesity (^29^). The inflammatory response, particularly within adipose tissue (^30^), plays a crucial role in obesity, as adipose tissue is known for producing inflammatory factors that can provoke chronic low-grade inflammation (^31^). This inflammatory response can activate pathways such as the NF-κB pathway, promoting the release of inflammatory factors such as tumor necrosis factor α (TNF-α) and interleukin-6 (IL-6). These factors can influence insulin sensitivity, fat metabolism, and energy balance, ultimately contributing to obesity development (^32^). This study identified cg18770216 (associated with the ITGB5 gene) as a regulator of obesity. ITGB5 encodes the integrin β5 subunit, which is involved in cell communication and signal transduction. Methylation in the ITGB5 gene may impact the inflammatory response and adipocyte differentiation, affecting fat metabolism and deposition (^33^). However, further research is necessary to elucidate the specific role of ITGB5 in obesity, including its involvement in cell signaling pathways, expression and regulation in adipose tissue, and interactions with other molecules that regulate inflammation and fat metabolism.

Future studies could explore alternative interventions for obesity and metabolic diseases. For example, intense intermittent exercise combined with thyme extract, as suggested in a previous review, may improve metabolic parameters related to obesity. In their research, 8 weeks of intense intermittent exercise with thyme extract was shown to affect the expression of the apoptosis indicators Bax and p53 in liver tissue and insulin resistance in type 2 diabetic rats, which implies possible positive effects on obesity-related metabolic disorders. Additionally, physical activity has various effects on health-related outcomes, as summarized in another review. Although that review focused mainly on cancer prevention, recovery, and recurrence, physical activity plays a crucial role in obesity prevention and treatment. Further research on its specific role in obesity-related metabolic regulation is warranted. These alternative approaches could provide new perspectives and strategies for combating obesity and its associated metabolic diseases.

This study has several limitations that warrant consideration. First, our findings demonstrate associations between methylation changes and obesity, but the cross-sectional design precludes causal inferences. While we hypothesize that methylation may influence obesity pathogenesis, it is equally plausible that obesity-related metabolic changes drive epigenetic modifications or that unmeasured confounders mediate both processes. Second, the sample size for the genome-wide methylation analysis (4 patients with obesity vs. 3 controls) was small, potentially limiting the statistical power and generalizability. Although the targeted validation cohort included larger groups (35 patients with obesity vs. 34 controls), the homogeneity of the young male population from a single geographic region may restrict broader applicability. Third, methylation was measured in peripheral blood, which may not fully reflect epigenetic patterns in metabolically active tissues. Fourth, despite correction for multiple testing, the exploratory nature of genome-wide analyses increases the risk of false-positive findings. The functional validation of candidate genes in relevant tissues or cellular models is also lacking. Fifth, the modest effect size of the correlation between BMI and methylation suggests that additional biological or environmental factors may contribute to obesity pathogenesis. Beyond that, we focused on single CpG site methylation levels rather than mean DNA methylation (DNAm) levels across DMRs for correlation with BMI. Future studies integrating regional methylation changes may further elucidate the epigenetic landscape of obesity. Finally, the cross-sectional design precludes causal inferences. Future studies incorporating longitudinal designs, Mendelian randomization, and intervention-based approaches are essential to disentangle causality.

In conclusion, the onset of adolescent obesity involves multiple mechanisms and factors. Our study revealed differential methylation sites and genes associated with obesity, highlighting LGI1, DIO3, and ITGB5 as potential contributors to obesity development. Future investigations can elucidate the specific mechanisms of these genes and their interactions with other obesity-related factors, offering a more in-depth understanding and potential therapeutic targets for obesity prevention and treatment.

## Data Availability

datasets related to this article will be available upon request to the corresponding author.
